# Breast Cancer Complicated by Cardiac Tamponade in a Patient With Neurofibromatosis Type 1

**DOI:** 10.7759/cureus.34095

**Published:** 2023-01-23

**Authors:** Narek Hakobyan, Nosakhare Ilerhunmwuwa, Henry O Aiwuyo, Ephrem Sedeta, Ifeanyi Uche, Mustafa Wasifuddin, Jamal C Perry

**Affiliations:** 1 Internal Medicine, Brookdale University Hospital Medical Center, Brooklyn, USA; 2 Medicine, Brookdale University Hospital Medical Center, Brooklyn, USA

**Keywords:** cardiac tamponade, neurofibromatosis type 1, invasive ductal cell carcinoma, recurrent pericardial effusion, neurofibromatosis type 1 (nf-1), breast cancer

## Abstract

Pericardial effusion may occur as a result of malignant pericarditis, which may in turn result in cardiac tamponade. This paper reports on a rare case of cardiac tamponade that occurred in an African American patient with breast cancer and neurofibromatosis. Herein, we present a case of a 38-year-old woman with neurofibromatosis type 1 (NF1) and breast cancer. She presented with sudden shortness of breath and hypotension. Computed tomography of the chest and an echocardiogram confirmed the presence of cardiac tamponade. Symptomatic relief was obtained following an emergency pericardiocentesis. The patient experienced a recurrence of symptomatic pleuro-pericardial effusion, requiring repeat therapeutic pericardiocentesis and thoracocentesis. To eliminate accumulating fluid, an indwelling drain was placed. The clinical condition of the patient, however, continued to deteriorate and she expired a few days after admission. When patients with breast cancer present with dyspnea, clinicians should maintain a high index of suspicion of cardiac tamponade; urgent imaging should be performed to exclude tamponade. Further research is needed to identify the factors that predict cardiac tamponade in breast cancer patients as well as the optimal treatment for the condition. It is also necessary to examine the relationship between a history of neurofibromatosis and cardiac tamponade.

## Introduction

Breast cancer has been postulated to be the most commonly occurring cancer in the United States (US) in 2022 [1]. According to the American Cancer Society, it accounts for over 51% of all new cancer diagnoses and contributes as much as 7% to cancer deaths in the US [1]. It is known to affect all genders and has been shown to be increasing in incidence over the past couple of years [1,2]. As with other growing tissues in the human body, it is also at risk of transforming into abnormal cells that have the propensity of becoming tumorigenic [3]. They can transform into benign or malignant tumors. Tumors of the breast may locally invade adjoining tissues and lymph nodes or metastasize to distant areas in the body [4]. The brain, lungs, liver, and bones are the most common sites of metastatic breast cancer [5]. Malignant pleural effusion occurs in approximately 7-11% of patients with breast cancer [6], with pleural effusion being the first sign of metastatic disease in 43% of these patients [7]. Pericardial effusion is only seen in 12-25% of patients who suffer from metastasis to the pericardium, and only a small percentage develop tamponade [8]. As a result, limited cases of cardiac tamponade associated with breast cancer have been reported. While breast cancer is known to spread to several organs within the body, pericardial involvement remains an unusual finding. The reason for this has not been fully studied; however, there exist isolated case reports of these occurrences among patients with breast cancer. Aside from the traditional risk factors for breast cancer development, there may be other genetic mechanisms that may be implicated in the development of this unique kind of pericardial involvement. As with all cancer diagnoses, the need to pay emphasis on prevention and early detection cannot be overemphasized.

Neurofibromatosis type 1 (NF1) is a fully penetrant autosomal dominant disorder with a prevalence of 1:5000, which is one of the neurocutaneous syndromes [9,10]. It is characterized by the development of neurofibromas along neural tissues of the body affecting the skin and the nervous system. The gene responsible for this disease is a tumor suppressor gene that antagonizes the effect of an oncogene called Ras [11]. Mutations in this tumor suppressor gene have been found to predispose patients to a wide range of cancers. There is growing evidence that breast cancer may be linked with NF1 [12-14], but more research needs to be done to establish a strong genetic association. Using meta-analysis, Suarez-Kelly et al. showed that women with NF1, who are less than 50 years old, have a five-fold increased risk of breast cancer, have a more severe disease presentation, and are more likely to die from the disease [15]. Tumors with NF1 pathology included both adenocarcinomas (36, 60%) and squamous cell carcinomas (10, 17%) [16]. We describe a 38-year-old African American woman with metastatic breast cancer and a history of NF1 who presented in cardiac tamponade.

## Case presentation

A 38-year-old female presented to the emergency room (ER) with new-onset shortness of breath associated with pleuritic chest pain. Medical history was significant for hypertension, NF1, pulmonary embolism, and stage IV estrogen positive/progesterone negative/human epidermal growth factor receptor-2 (HER2) negative breast cancer of the right breast for which she had undergone lumpectomy plus chemotherapy and at the time of admission was undergoing radiotherapy.

In the emergency department, the patient was found to be hypoglycemic (21 mg/dL) and the bedside ultrasound showed pericardial effusion along with bilateral pleural effusions. Lab findings were remarkable for hemoglobin (Hgb) of 5.7 g/dL, hematocrit (HCT) of 17.3%, and platelets of 93,000 mcL. She became hypotensive with a blood pressure of 70/40mmHg requiring placement of a right femoral line and administration of 500 mL of bolus normal saline and two units of packed red blood cells. She was transferred to the intensive care unit for more vigorous monitoring and treatment.

**Figure 1:**
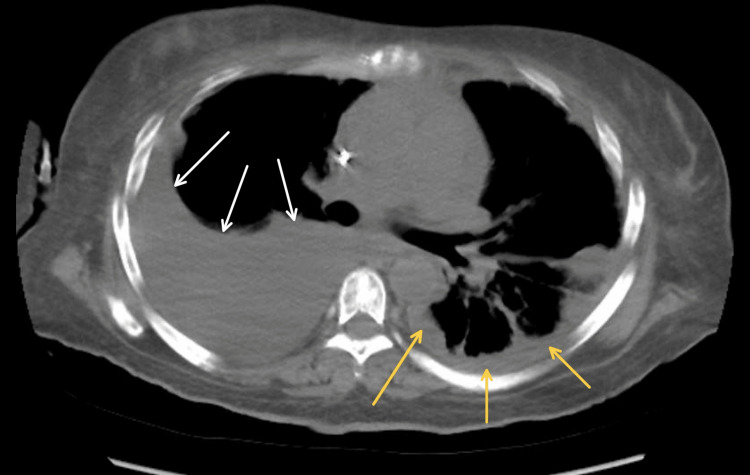
Large right-sided pleural effusion (white arrows) and left-sided pleural effusion (yellow arrows)

The Hgb increased to 9.1 g/dL following the blood transfusion. The suspicion of gastrointestinal bleeding was high since she did not exhibit any visible signs of bleeding. Due to her shortness of breath and inability to lay supine, the risk of diagnostic evaluation outweighed the benefits of performing an esophagogastroduodenoscopy and/or colonoscopy, thus were not utilized. CT of the chest, abdomen, and pelvis was done in addition to the symptomatic management of anemia. She was transfused with packed RBC to keep Hgb > 7 g/dL. Large right pleural effusion, moderate left pleural effusion, and left pericardial effusion measuring up to 3.8 cm in thickness were noted on the CT scan (Figure 1).

**Figure 2:**
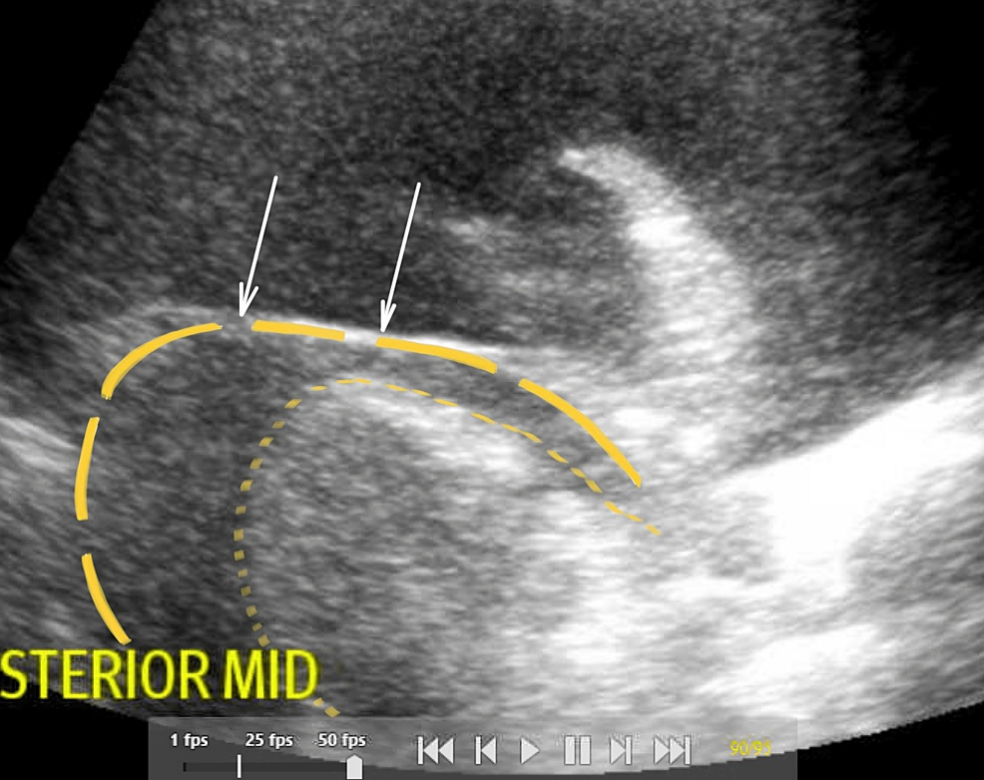
Large pericardial effusion (white arrows) encompassing the heart in subxiphoid

A follow-up echocardiography (ECHO) was performed revealing pericardial tamponade, shown in Figure 2, requiring pericardiocentesis. Subsequently, thoracentesis and pericardiocentesis were performed. A total of 1 liter of serosanguinous fluid was removed from the right chest and 500 mL of bloody fluid from the left chest. Pericardiocentesis yielded 750 mL of dark sanguineous fluid, which was sent for cytology yielding malignant scattered aggregates of carcinoma with moderate to marked atypia with mitosis consistent with metastatic carcinoma from primary breast cancer. The patient had a chest tube placed to suction for an iatrogenic pneumothorax. The patient’s family came to an agreement for do not resuscitate/do not intubate (DNR/DNI) status and palliative care was consulted for end-of-life care. The patient was found unresponsive and was pronounced deceased four days after admission.

## Discussion

An invasive local neoplasm or metastatic neoplasm that has spread to the pericardium can cause pericardial effusion, which represents the extension of malignant cells into the pericardium. It has been determined that patients with previously undiagnosed or newly diagnosed malignancies have a mean survival rate of four to six months after being diagnosed with malignant pericardial effusions [17-19]. Two-thirds of cancer patients, however, suffer from pericardial effusion because of non-malignant processes, such as chemotherapy, radiation therapy, and infection [20]. The presence of neoplastic cells in the pericardial fluid is an independent prognostic indicator of mortality [21,22].

Cardiac tamponade occurs when fluid accumulates around the heart and leads to pericardial compression and low cardiac output. As the volume and pressure increase, the pericardium's elastic limit is eventually overcome by the progressive increase in fluid. This causes external pressure to be exerted on the chambers of the heart leading to rapid equalization of diastolic pressures and loss of ventricular interdependence. A high level of pressure within the pericardial space causes compression of the cardiac chambers, decreases the flow of venous blood to the heart, and impairs diastolic function [23]. A decrease in the volume of the cardiac chambers has the effect of filling the chambers with less blood, ejecting a smaller amount of blood with each contraction. This results in a decrease in cardiac output and blood pressure. A tamponade is defined as a state in which systemic perfusion does not meet normal levels and venous return to the heart is impaired. It is common for patients to experience symptoms of dyspnea, chest discomfort, and fatigue during the early stages of the disease. Symptoms of tamponade physiology result in patients deteriorating and developing shock [24]. Our patient had dyspnea and pleuritic chest pain consistent with pericardial disease.

In addition to being difficult to treat, malignant pericardial effusions have limited treatment options. The use of emergent pericardiocentesis in patients suffering from cardiac tamponade is recommended to avoid shock and death [25]. Our patient's ECHO study showed massive pericardial effusion with cardiac tamponade warranting emergency pericardiocentesis, which is the management modality for pericardial tamponade. Pericardiocentesis is the primary procedure to diagnose and treat large effusions without tamponade. Performing pericardial fluid analysis confirms the neoplastic nature of the effusion, delaying its recurrence and adverse effects on the patient's condition [9]. When the effusion has been associated with a locally invasive tumor, definitive treatment of underlying cancer improves the outcome [25,26]. Pericardiocentesis can be performed routinely to prevent recurrences, or intrapericardial injection of cytostatic and sclerosing agents can be given, or a pericardial window may be surgically created [27-29]. Our patient developed symptomatic relief after the effusion was drained but she had deaccumulation of the fluid as well as pleural effusion.

Reducing symptoms is more important than treating the underlying disease when preventing pericardial effusions from recurring. Palliative interventions, such as the creation of a surgical pericardial window, improve the quality of life of patients suffering from advanced malignancies and decrease the number of days spent in the hospital. Although palliative interventions have been shown to improve quality of life in some select groups of patients, our patient did have a short in-hospital course with very severe symptoms that prevented any advanced strategies for long-term palliation to be administered. So, we recommend that physicians identify patients who are likely to experience fatal presentations of cardiac tamponade and provide expedited palliative surgical measures well in advance.

Our patient's history of NF1 is also concerning as there is good literature in support of an association with breast cancer. Information about the genetics of the breast cancer hormone receptor status and BRCA testing is necessary for proper characterization. Unfortunately, these reports were not available in the patient's charts and the patient was already in the end-of-life pathway. The Ras association has been linked to the development of breast cancer in a patient with NF1. We do not know as of now if Ras proto-oncogene is a surrogate for the early development of breast cancer; however, our patient was a young African American woman who had NF1, and subsequently developed breast cancer with pericardial metastasis, which is a very rare occurrence. We recommend that clinical oncologists risk stratify patients who are at increased risk of mutagenesis and consider them for more aggressive management during the initial stages of therapy. The need to consider these patients with NF1 for early screening for breast and other gynecologic malignancies is therefore imperative in our opinion.

## Conclusions

Malignant pericardial tamponade is a life-threatening complication of breast cancer. Its propensity to recur in patients with cancer confers high morbidity and mortality. The very occurrence of this complication is indicative of a poor prognosis for patients. At-risk patients should, therefore, be monitored closely by physicians for malignant cardiac tamponade. Cancers of the lung, breast, hematological system, and gastrointestinal system are among the most common malignancies implicated in this syndrome. Pericardiocentesis should be performed urgently in cases of large effusions with or without pericardial tamponade as a diagnostic and therapeutic intervention. The treatment of cardiac tamponade is generally palliative. The goal of treatment is to prevent progression to cardiac tamponade and to improve quality of life through the alleviation of symptoms.
